# The value of CT imaging and CRP quotient for detection of postbariatric complications

**DOI:** 10.1007/s00423-020-01986-w

**Published:** 2020-09-17

**Authors:** Anna Duprée, Jocelyn de Heer, Michel Tichby, Tarik Ghadban, Oliver Mann, K. Grupp, Hans O. Pinnschmidt, Jakob R. Izbicki, Stefan Wolter

**Affiliations:** 1 Department of General, Visceral and Thoracic Surgery, University Medical Center Hamburg-Eppendorf, Martinistr. 52, 20246 Hamburg, Germany; 2 Center for Radiology and Endoscopy, Department of Interdisciplinary Endoscopy, University Medical Center Hamburg-Eppendorf, Hamburg, Germany; 3 Department of Plastic Surgery, University Medical Center Hamburg-Eppendorf, Hamburg, Germany; 4 Center for Experimental Medicine, Institute of Medical Biometry and Epidemiology, University Medical Center Hamburg-Eppendorf, Hamburg, Germany

**Keywords:** Complications, Bariatric surgery, CT scan, CRP quotient

## Abstract

**Background:**

The diagnosis of major complications seems to be more challenging in obese patients. We aimed to show the relevance of routinely assessed clinical and paraclinical parameters as well as the relevance of CT scans in the diagnosis of major complications after bariatric procedures.

**Methods:**

All patients who underwent operations (primary or revisional) in a 3-year period were retrospectively studied after bariatric surgery with a specific focus on the routinely assessed clinical parameters (tachycardia, temperature), paraclinical parameters on postoperative day (POD) 1 and 3 (C-reactive protein (CRP), leukocytes), and additional computed tomography (CT) scan results for the diagnosis of leakage, bleeding, intraabdominal abscess, superficial abscess, and other complications.

**Results:**

A total of 587 patients were examined. In this cohort, 73 CT scans were performed due to suspected intraabdominal or pulmonary complication according to our hospital standard operating procedure. In total, 14 patients (2.4%) had a major complication (Clavien-Dindo grade IV/V). Of those, 10 patients (1.7%) had postoperative leakage. While the correct leakage diagnosis was only found in 33% of the patients by CT scan, the overall specificity of CT as a diagnostic tool for all kinds of complications remained high. Especially for abscess detection, CT scan showed a sensitivity and specificity of 100%. Multivariate analysis showed a significantly higher risk of leakage development characterized by a doubling of postoperative CRP level (odds ratio 4.84 (95% confidence interval 2.01–11.66, *p* < 0.001)). To simplify the use of CRP as a predictive factor for the diagnosis of leakage, a cut-off value of 2.4 was determined for the CRP quotient (POD3/POD1) with a sensitivity of 0.88 and a specificity of 0.89.

**Conclusion:**

CT diagnostic after bariatric surgery has a high positive predictive value, especially for intraabdominal abscess formation. Nevertheless, CT scan for the diagnosis of leakage has a low sensitivity. Thus, a negative CT scan does not exclude the presence of a leakage. Using the described CRP quotient with a cut-off of 2.4, the risk of early leakage can be easily estimated. Furthermore, in any uncertain case of clinically suspected leakage, diagnostic laparoscopy should be performed.

## Introduction

Bariatric surgery can be performed with low complication rates and is known to be safe. Staple line leakage is reported in up to 3.8% of cases [1, 2] and has been identified as the strongest risk factor for mortality [3]. Early diagnosis and treatment are mandatory for good outcomes. The diagnostic method of choice varies according to the institution and surgeon. When bariatric surgery was first being performed, each patient underwent routine upper gastrointestinal fluoroscopy (UGI); currently, with increases in experiences only in cases of adverse events, radiologic imaging is performed. Computed tomography (CT) scans are commonly used for the diagnosis of complications [4], although the diagnostic significance is known to be poor in obese patients. Nevertheless, few studies have reported controversial outcomes concerning the sensitivity of CT scans in obese subjects [5–7].

The aim of our study was to evaluate the diagnostic value of CT scans in the diagnosis of postoperative complications in obese individuals, with a specific focus on different events, such as leakage or abscess. Furthermore, we wanted to establish a pathway for diagnosis in order to assess clinical parameters using the CRP quotient.

## Methods

A retrospective analysis of a 3-year cohort of patients who underwent bariatric surgery was performed. Certified bariatric surgeons (> 100 bariatric procedures) at a bariatric center of excellence in Germany performed all surgical procedures. Data was collected prospectively and were assigned anonymously to the bariatric database. No patients were excluded within the mentioned period. A total of 587 patients in the mentioned period were screened for surgical complications, such as leakage, bleeding, abscess, and wound infection. Performed CT scans in those patients were collected and evaluated. For statistical analysis, surgical complications were categorized into groups: leakage (anastomotic insufficiency/staple line leakage), bleeding, intraabdominal abscess, superficial abscess, and other major adverse events (such as ischemia, colonic perforation, and spleen rupture). The distinction into the groups was made by the final diagnosis. Those were correlated with clinical and paraclinical parameters, such as C-reactive protein (CRP), leukocytes, temperature, and heart frequency. Blood samples were collected as a part of routine measurements at our central laboratory preoperatively and on postoperative day (POD) 1 and 3 according to the hospital standard. All detected adverse events within 30 days after surgery were documented. CT scans with oral and intravenous contrast dye were carried out in patients with clinically suspected complications (also for pulmonary complications, i.e., pulmonary embolism) or highly elevated blood parameters according to the hospital standard. In any doubt, endoscopy or diagnostic laparoscopy was performed to confirm or discard the suspected complication. For statistical analysis, surgical complications were categorized into groups: leakage, bleeding, intraabdominal abscess, superficial abscess, and other major adverse events (such as ischemia, perforation, and spleen rupture). The distinction into the groups was made by the final diagnosis. Patients without any complication were subgrouped as a control group.

## Statistical analysis

Mean (with standard deviation) and median (range) values were calculated for normally and non-normally distributed continuous data, respectively. The normality of the distribution of continuous variables was assessed based on histograms. Right-skewed variables were transformed to their logarithm prior to subsequent analyses. Univariate and multivariate logistic regression analyses were performed to examine potential predictors for various types of complications. In the univariate analyses, individual independent variables were singly forced into the regression equation (method “enter”). A stepwise forward approach was used for variable selection in the multivariate analyses to avoid overparameterization of the regression models because the number of complications was relatively low in relation to the total number of patients. The predictive capacity of CRP for early complications and leakage was further assessed using a receiver operating characteristic (ROC) curve analysis. The cut-off value with the best sensitivity and specificity for the detection of complications was determined as the maximum Youden index (sensitivity − (1 − specificity)) obtained from the various values of a variable. The ROC curve analysis results were reported as the area under the curve (AUC) with corresponding 95% confidence intervals (CIs) and *p* values, testing the null hypothesis that the area under the curve would be equal to 0.5. To determine the relations among nominal variables, *χ*^2^ tests were used. For comparisons of the two groups, independent-sample Student’s *t* tests were employed for continuous variables. The significance level was set at *α* = 0.05. All tests were two-tailed. All statistical analyses were performed with SPSS version 25 (IBM Corp.)

## Results

A total of 587 patients underwent bariatric surgery. Of these, 215 patients were male (36.6%), and 372 were female (63.4%). The median BMI was 49.9 kg/m^2^ (range 35.0–110 kg/m^2^). The median age of all patients was 44.4 years (range 14–74 years). Overall, 257 (43.8%) patients underwent sleeve gastrectomy (SG), and 297 (50.6%) underwent Roux-en-Y gastric bypass (RYGB), including redo procedures. The remaining 5.6% of patients received other bariatric procedures, such as gastric band implantation (LAGB) and explantation, as well as biliopancreatic diversion ± duodenal switch (BPD/DS). In 562 (95.7%) patients, the procedure was completed laparoscopically. A total of 110 (18.7%) patients had undergone a previous bariatric procedure: 49 had undergone SG (44.5%), 26 gastric banding (23.6%), 18 LAGB removal (16.4%), 10 RYGB (9.1%), and 7 duodenal-pacer-implantation and explantation (6.4%).

In 587 of the bariatric procedures performed, 14 patients (2.4%) had a major complication (Clavien-Dindo grade IV/V). Of those, 10 patients (1.7%) had postoperative leakage, 7 after sleeve gastrectomy (1.2% of total), and 3 after RYGB (0.5% of total). Four patients had other reasons, such as mesenteric ischemia (*n* = 2), colonic perforation (*n* = 1), and spleen rupture (*n* = 1). Five patients (0.6%) had intraluminal or extraluminal bleeding according to Clavien-Dindo grade II/III. In 6 patients (1.0%), intraabdominal abscess was detected (Clavien-Dindo grade III), and in another 11 patients (1.9%), superficial abscess and wound complication occurred (Clavien-Dindo grade I/IIIb). Complications could be managed successfully in the majority of patients by revisional surgery (leakage, bleeding, superficial abscess), interventional abscess drainage, or endoscopic procedure (bleeding, leakage). Two patients died during the 30-day observational period (0.3%). Both of them were treated by surgical revision. One of them had leakage after sleeve gastrectomy with fulminant multi-organ failure, and the other one died due to mesenterial ischemia.

## Diagnostic value of the CT scan

CT scans were performed in any case of clinical suspicion for a complication. Most of the patients presented with impaired general condition and elevated parameters of infection. Only few showed abdominal pain. In case of bleeding, normally, an endoscopy was the first diagnostic and therapeutic choice.

In total, 73 (12.4%) patients underwent CT scans for suspected complication. Of these, 51 (69.8%) patients were female, and 22 (30.1%) patients were male. The median BMI was 48.2 kg/m^2^ (36.0–68.0 kg/m^2^), and the median age was 43.6 years (19–68 years). A total of 45 (61.6%) patients in the CT group underwent RYGB, and 25 (34.3%) patients underwent SG. Three had gastric band removal or reimplantation. Eighteen (24.7%) patients had undergone previous bariatric surgery. Redos and revisional surgery did not show higher incidence of complications.

A total of 59 (80.8%) patients in the CT group had at least one obesity-related comorbidity while 404 (78.8%) patients in the non-CT group.

In 83.6% (*n* = 61) of the patients, the diagnosis according to the CT scan was correct. In 15.1% (*n* = 11), the CT scans showed overall false-negative results, resulting in an overall sensitivity of 0.54 and a specificity of 0.98 (Table 1). CT performance for the diagnosis of leakage was poor. However, in patients with intraabdominal abscess formation, the CT scan resulted in a correct diagnosis in 100% (*n* = 5), with no false-negative results, leading to a positive predictive value (PPV) of 1. The overall specificity of CT as a diagnostic tool remains high for every clinical evaluation. The sensitivity is highest in cases of intraabdominal (1) or superficial abscess (0.75), each with a PPV of 1 (Table 1).

**Table 1:** The use of CT for the diagnosis of bariatric complications

CT	Total	Leakage	Bleeding	IA	SA	Other
r-p	13	3	0	5	3	2
r-n	48	64	71	68	69	68
f-p	1	0	0	0	0	0
f-n	11	6	2	0	1	3
sens	0.54	0.33	NA	1	0.75	0.4
spec	0.98	1	0.97	1	1	1
PPV	0.92	1	0	1	1	1
NPV	0.81	0.91	1	1	0.98	0.95

*CT*, computed tomography; *IA*, intraabdominal abscess; *SA*, superficial abscess; *r-p*, right-positive; *r-n*, right-negative: *f-p*, false-positive; *f-n*, false-negative; *sens*, sensitivity; *spec*, specificity; *PPV*, positive predictive value; *NPV*, negative predictive value

## Paraclinical parameters

The paraclinical parameters in patients with complications were examined (Table 2). Of those, only CRP showed significance in the univariate and multivariate analysis.

**Table 2:** Paraclinical parameters for bariatric complications

	POD1				POD3			
	CRP (mg/l)	Leu (Bn/l)	HR (bpm)	Temp (C°)	CRP (mg/l)	Leu (Bn/l)	HR (bpm)	Temp (C°)
Total	51 [6–301]	13.1 [7.6–22.9]	80 [55–120]	36.5 [35.2–38.0]	112 [13–525]	12.1 [6.1–22.1]	82 [55–110]	36.6 [35.5–38.2]
Control	42 [5–260]	11.3 [4.4–69.0]	80 [42–119]	36.3 [34.6–38.3]	59 [5–408]	9.5 [3.5–23.5]	80.5 [40–120]	36.6 [35.3–38.2]
Leakage	28 [18–165]	11.9 [8.2–22.3]	72 [55–120]	36.3 [35.2–37.1]	165 [27–525]	14.5 [8.0–17.2]	90 [64–110]	36.8 [36.0–38.2]
Bleeding	68 [6–77]	10.7 [10.4–19.9]	80 [66–105]	36.1 [35.7–37.2]	83 [82–100]	10.1 [7.7–17.8]	85 [71–107]	36.5 [36.1–37.4]
IA	50 [45–207]	12.6 [7.6–18.4]	81.5 [56–113]	36.5 [36.0–37.2]	78 [39–196]	12.1 [6.7–12.9]	85 [55–93]	36.6 [36.2–37.1]
SA	95 [6–301]	13.1 [8.2–18.1]	80 [71–97]	36.4 [36.0–37.1]	93 [48–326]	9.8 [6.1–16.1]	86.5 [70–98]	36.3 [36.0–36.7]
Other	118 [7–190]	10.3 [7.9–13.2]	85.5 [70–95]	36.5 [36.0–36.8]	90 [13–117]	10.6 [9.2–11.4]	85.5 [80–96]	36.3 [35.5–36.8]

Values are shown as the mean [range]; *POD*, postoperative day; *IA*, intraabdominal abscess; *SA*, superficial abscess; *CRP*, C-reactive protein; *Leu*, leucocytes; *HR*, heart rate; *bpm*, beats per minute; *Temp*, temperature; *°C*, Celsius

**Fig. 1:**
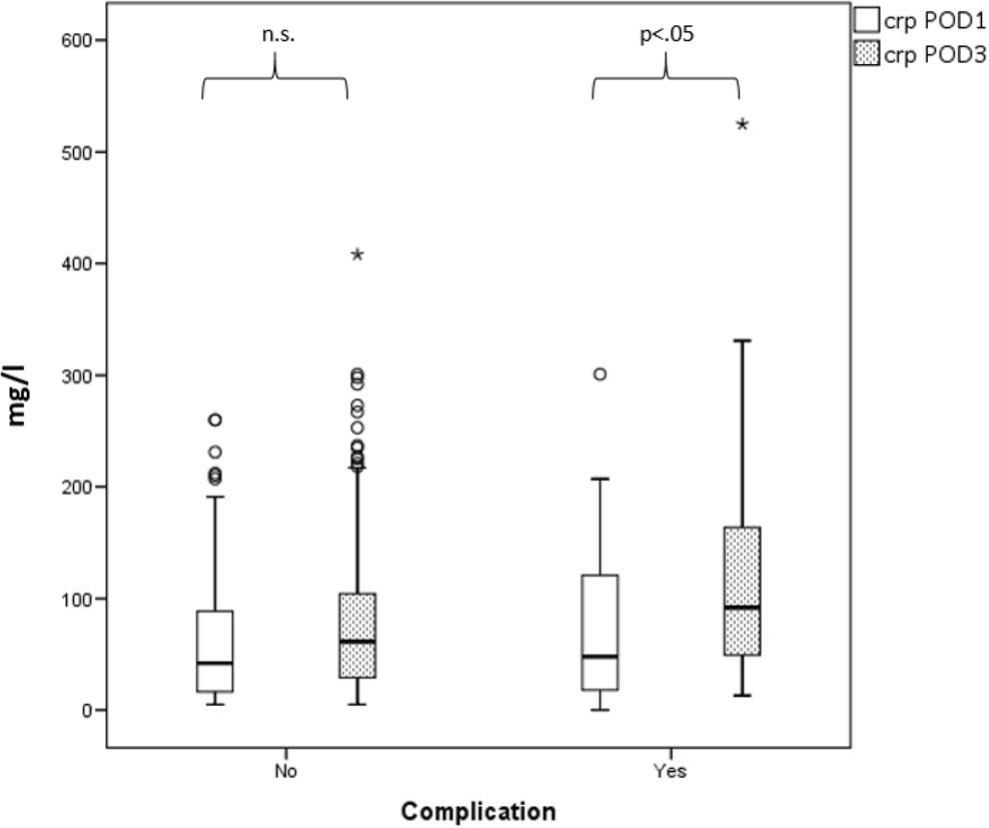
Box plot of CRP for any type of 30-day complication after bariatric surgery.CRP in mg/l is shown for complications (right) and for no complications (left) on POD 1 and POD 3. CRP, C-reactive protein; POD, postoperative day; n.s., not significant

**Fig. 2:**
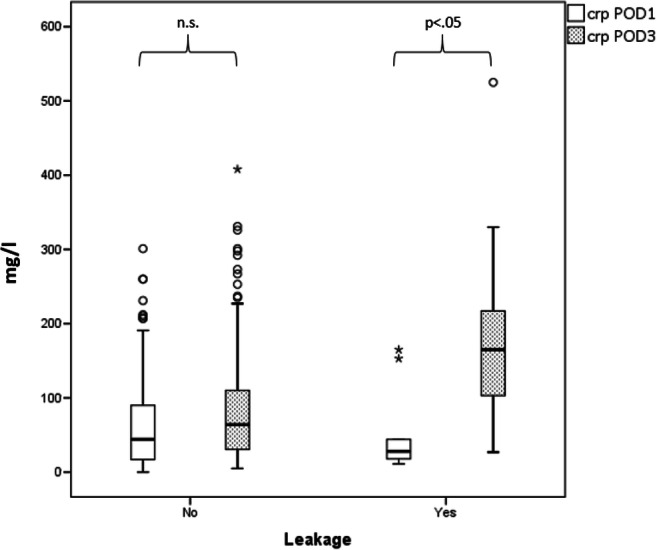
Box plot of CRP for leakage after bariatric surgery. CRP in mg/l is shown for leakage (right) and for no leakage (left) on POD 1 and POD 3. CRP, C-reactive protein; POD, postoperative day; n.s., not significant

The median CRP for the presence of any kind of complication was 51 mg/l on POD1 and 112.5 mg/l on POD3. CRP in the control group was 42 mg/l on POD1 and 59 mg/l on POD3. Thus, CRP on POD3 was significantly higher in the complication group (Fig. 1) and for a diagnosis of leakage alone (Fig. 2). The univariate and multivariate analyses of the paraclinical parameters in the other complication groups showed no significance.

## Leakage

Since the diagnostic uncertainty in our cohort primarily relates to the diagnosis of leakage, we have examined this complication separately.

### CT scan for the diagnosis of leakage

Of the 10 patients with a leakage (1.7%), 9 had had CT scan. Of these, only 33% (*n* = 3) were diagnosed correctly, while the remaining 6 patients had false-negative results. Accordingly, sensitivity was lowest (0.33) for detection of leakage, while the specificity was 1. In all of these patients, a diagnostic laparoscopy was performed according to the hospital standard due to their clinical condition.

### Paraclinical parameters for the diagnosis of leakage

In the univariate analysis, the doubling of CRP on POD3 in the leakage group showed an OR of 2.5 (95% confidence interval [CI] 1.25–4.88, *p* = 0.009), whereas in the multivariate analysis, the doubling of CRP resulted in an OR of 4.84 (95% CI 2.01–11.66, *p* = 0.000). Furthermore, the leucocyte elevation of 1.0 Bn/l on POD3 compared with the count on POD1 showed significant results in the univariate analysis, resulting in an OR of 1.26 (95% CI 1.08–1.47, *p* = 0.03). Using the Youden index (YI) in the ROC analysis, a cut-off value of 14.45 Mrd/l on POD3 was determined (sensitivity 0.56, specificity 0.89; *p*_AUC_ = 0.004). The area under the curve (AUC) of the leucocyte count on POD3 was 0.5, thus not reaching any statistical significance.

### CRP for the diagnosis of leakage

Using the YI, the cut-off value for CRP on POD3 was 147 mg/l (YI: 0.43, sensitivity 0.556, specificity 0.87; *p*_AUC_ = 0.013). CRP on POD1 showed an AUC of 0.4, resulting in no discrimination capacity.

**Fig. 3:**
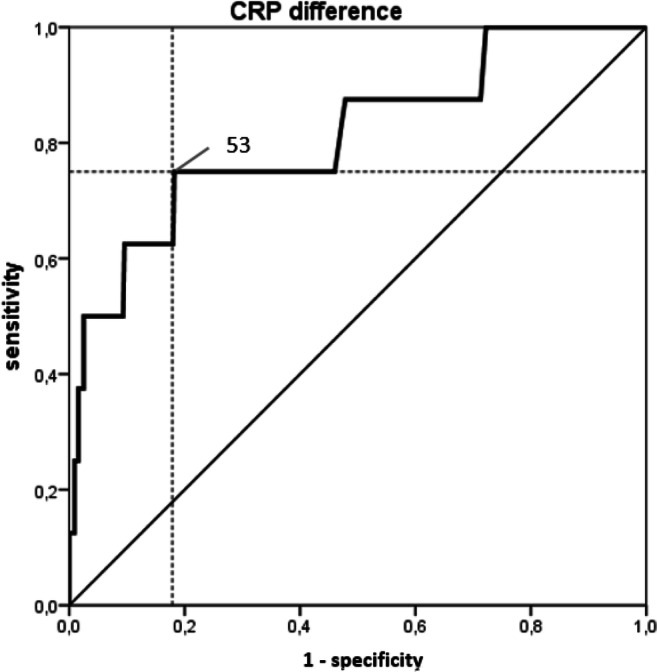
ROC curve of the Youden Index for the CRP difference. CRP difference (POD3-POD1) showed high diagnostic value with an AUC of 0.81 (95% CI 0.64–0.98, *p*_AUC_ = 0.003). ROC, receiver operating characteristic; CRP, C-reactive protein; POD, postoperative day; AUC, area under the curve

**Fig. 4:**
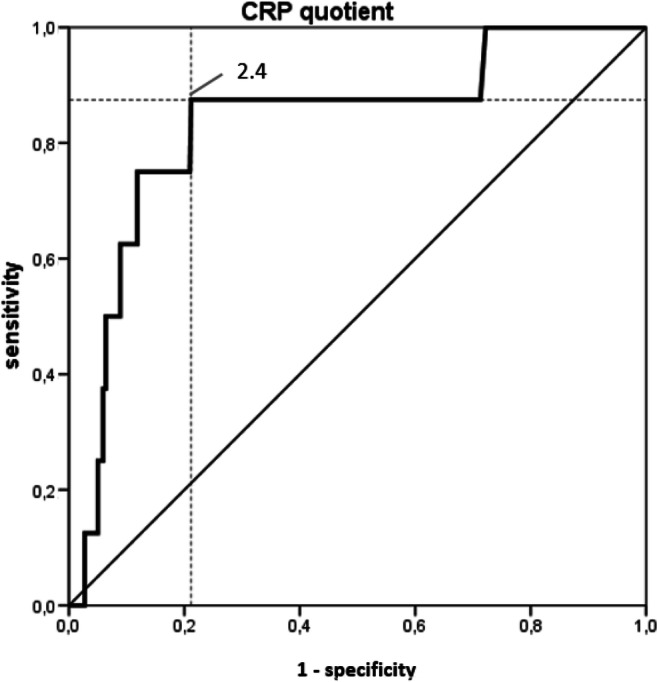
ROC curve of the Youden Index for the CRP quotient. CRP quotient (POD3/POD1) showed high diagnostic value with an AUC of 0.83 (95% CI 0.68–0.98, *p*_AUC_ = 0.001). ROC, receiver operating characteristic; CRP, C-reactive protein; POD, postoperative day; AUC, area under the curve

To simplify the use of CRP as a predictive factor for the diagnosis of leakage, we tested the CRP difference (POD3-POD1) (Fig. 3) and the CRP quotient (POD3/POD1) (Fig. 4). Both the difference and quotient showed high diagnostic value with AUCs of 0.81 (95% CI 0.64–0.98, *p*_AUC_ = 0.003) and 0.83 (95% CI 0.68–0.98, *p*_AUC_ = 0.001), respectively. Thus, using the YI, a cut-off value of 2.4 for the CRP quotient was determined with a sensitivity of 0.88 and a specificity of 0.89. For the CRP difference, a cut-off of 53 mg/l with a sensitivity of 0.75 and a specificity of 0.82 was defined.

## Discussion

The results of our study show that the use of CT scans for the diagnosis of leakage had a poor sensitivity of only 33%, and 67% showed false-negative results. Anyway, for other diagnosis, such as intraabdominal abscess formation, CT scan showed a specificity and sensitivity of 100%.

Although the best radiologic method for detecting complications following bariatric surgery is still controversial, selective CT scans with oral contrast agents seem to be superior to upper fluoroscopy with a reported sensitivity of 95% (95% CI 81.8 to 99.1) and a specificity of 100% (95% CI 93.1 to 100) for diagnosing postoperative leaks [5, 8]. However, many authors have discussed the diagnostic impact of CT scans. In a prospective evaluation of routine early CT scans, Lainas et al. showed a comparable low sensitivity of only 45.4% for the diagnosis of staple line leakage in sleeve gastrectomy as well as a specificity of 100% [8]. On the other hand, Kalff et al. reported the results of 66 CT scans, revealing a sensitivity of 89 to 100%, a specificity of 69 to 78%, a PPV of 39 to 50%, and an NPV of 97 to 100% for leakage diagnosis [9]. They conclude that a negative CT scan rules out leakage. Comparable with the result of Lainas et al., our results suggest that a negative CT scan does not exclude the presence of a leakage.

The use of CT for diagnoses in obese patients is known to be difficult. The quality of imaging is impaired because of truncation, cropping, and ring artifacts, as well as complex contrast agent dynamics [10]. Due to this uncertainty, even in highly experienced radiologic centers, several authors emphasize the value of clinical and paraclinical parameters of inflammation.

Tachycardia is known to be one of the earliest markers of a complicated course in bariatric surgery [11–13]. Hamilton et al. showed that 90% of patients with a leak demonstrated severe tachycardia (> 120 bpm.), and only 16% of patients in the control group demonstrated tachycardia. However, all of the patients with leakage and more than half of those in the control group were tachycardic (heart rate > 100 bpm) postoperatively [12]. Thus, elevated heart rate alone remains an uncertain marker with poor sensitivity. From our data, the mean heart rate in the case of leakage was found to be 76.6 ± 17.9 on POD1 and 87.7 ± 14.0 on POD3. Since tachycardia also occurs in many other postoperative situations, the use of this parameter alone remains inaccurate.

In a meta-analysis that included 7 studies [14–20], Bona et al. ascertained that CRP on POD1 was significantly higher in the case of complications (14.9 mg/l) than in the case of a normal postoperative course (4.3 mg/dl; *p* = 0.013) [21]. Similar results were observed in studies that determined CRP levels on POD2 (8.6 mg/dl vs. 23.7 mg/dl; *p* = 0.001) [21]. Thus, with the use of a Bayesian meta-analysis, the optimal CRP cut-off value on POD1, as a result of 5 studies and 1202 patients, was determined to be 6.1 mg/dl, reaching a sensitivity of 0.82 (95% CrI 0.71–0.91) and a specificity of 0.92 (95% CrI 0.71–0.99). Villard et al. described a CRP cut-off value ≥ 5 mg/dl on POD1 with a sensitivity of 27% and a specificity of 88% [16]. Accordingly, the sensitivity was very low. In our study, CRP on POD1 for any kind of complication was not significantly different from that of non-complicated cases and was lower in both groups than the described CRP cut-off value of 6.1 mg/dl (42 mg/l vs. 51 mg/l; n.s). On POD3, CRP in patients with complications was significantly higher than that in normal patients (113 mg/l vs. 59 mg/l; *p* > 0.001). Thus, in our opinion, the use of the CRP quotient (POD3/POD1) is useful because it takes into account the normal increase in CRP at the first POD due to operative trauma. Additionally, the CRP difference might be a useful tool to detect early leakage after bariatric surgery. However, the different units have to be noted (mg/l in this study, transformed in the discussion), making the quotient easier to work with than the difference. Nevertheless, both parameters showed high diagnostic value in the ROC analysis.

**Fig. 5:**
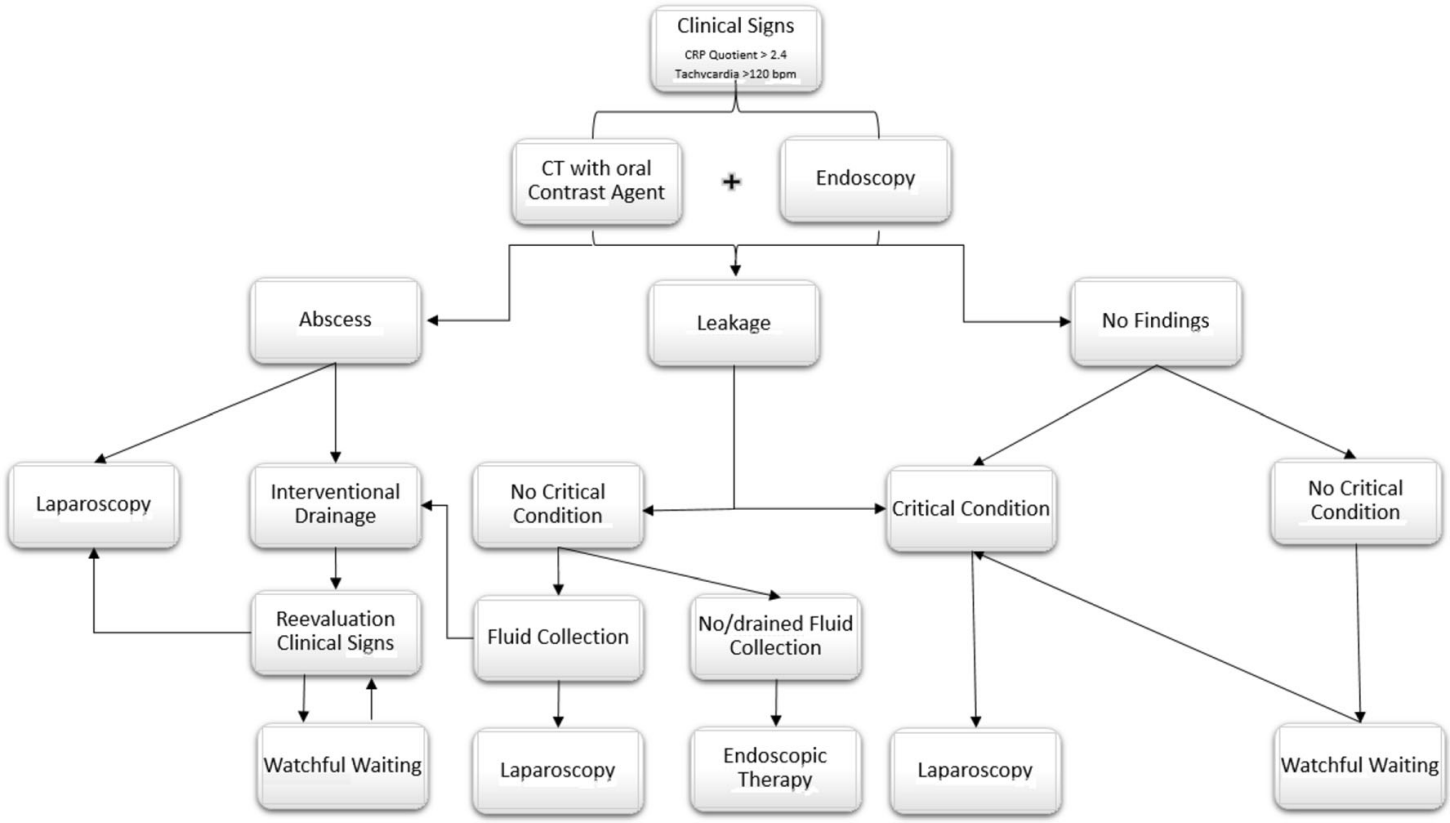
Internal clinical pathway for leakage management. The typical pathway for leakage management in our institution is shown. When there is any doubt, or in cases of clinical impairment with critical illness, diagnostic/therapeutic laparoscopy is performed. Depending on the intraoperative findings and overall constitution, laparotomy is recommended. In situations where patients are stable with no major intraabdominal fluid collection or when there is the possibility of sufficient drainage, endoscopic therapies are useful. Thus, we prefer Endo-sponge therapy. The main goal is drainage to avoid generalized peritonitis, whereas suturing of infected areas usually does not result in healing

Based on these single-center results, we developed a complication management pathway that has been used in our center of excellence (Fig. 5). To simplify the assessment of CRP changes postoperatively and to compensate for individual variation, we consider the quotient to be more useful than the cut-off value of the raw parameter alone.

## Conclusion

The use of CT for the diagnosis of leakage has a low sensitivity. However, in cases of intraabdominal abscess, CT is the diagnostic tool of choice. The specificity is high for every clinical evaluation. Thus, the value of CT scans is justified for detecting complications after bariatric surgery. With the use of the described CRP quotient with a cut-off value of 2.4, the risk of early leakage can be easily estimated. Therefore, this approach closes the gap in terms of the failure of CT scans. Furthermore, in any uncertain case of clinically suspected leakage, a diagnostic laparoscopy should be performed.
